# Intervention mechanism of healthcare service goods based on social welfare maximization in China

**DOI:** 10.1371/journal.pone.0214655

**Published:** 2019-03-29

**Authors:** Hao Li, Jinlin Li, Jingrong Zhu

**Affiliations:** Department of Management Science and Engineering, Beijing Institute of Technology, Beijing, China; Navrongo Health Research Centre, GHANA

## Abstract

In this paper, we aim to establish a mathematical model to design a maximizing social welfare intervention mechanism of healthcare service goods in China. The intervention mechanism is helpful to facilitate the adoption of the healthcare service goods. We consider a research problem that regulates the supply chain system for healthcare service goods by an intervention mechanism, and two intervention strategies composed of demand-growth strategy and subsidy strategy are used to the combination of intervention mechanism. Then this paper presents a new method based on fuzzy set and bilevel programming to design the intervention mechanism. To demonstrate the effectiveness of the proposed model, we conduct a case study for Wudang personalized health package and verify our model by the specific result analysis, the result indicates that our joint intervention mechanism is helpful to achieve the target and increase social welfare.

## Introduction

The global population is ageing rapidly due to a decline in fertility and an increase in life expectancy [1]. In fact, healthy bodies can contribute to their families and the society positively [2]. In 2016, guidance policy on promoting healthcare services goods is proclaimed by State Council of China, National Commission of China and other national ministries. Hence, local governments, scientific research institutions and healthcare industries has put much attention on healthcare service goods. Basic healthcare service goods covers diagnosis and treatment of common diseases, frequently-occurring traditional Chinese and western medicine, rational drug use, guidance of medical treatment path and referral appointment. In fact, promoting healthcare service goods is not only an important task of deepening the reform of the medical and health system, but also an important way to better safeguard the health of the people under the new situation. Healthcare service goods belong to public goods, which can be identified as goods with huge positive externalities. Public goods make it possible to enjoy the benefits for others although they do not pay for these goods, which is different from the property of private goods significantly. Furthermore, healthcare service goods are beneficial to extend expected lifetime and promote regional health development level. At present, the promotion of healthcare service goods is at the initial stage, and China medical insurance system has not covered healthcare service goods due to the system’s imperfection, it partially explain why patients are not willing to purchase healthcare service goods. In other word, it is necessary for policy makers to promote healthcare service goods by an effective intervention mechanism. As we all know, conventional hospital resource is not enough to meet the expectation of residents’ demand in China, however, the importance of healthcare service goods does not get a lot of attention, so establishing an intervention mechanism of healthcare service goods is extremely important and necessary. The rapid growth of China's economy has improved people’s living standards dramatically [3], with the growth of various health demand in China, healthcare service goods have emerged in large cities in recent years. Personalized health package, chronic disease management service goods, postpartum recovery service products, or vaccines used for prophylactic vaccination are well-known examples of healthcare service goods. In fact, the usage and promotion of healthcare service goods is beneficial to actual customers and majority of local residents remarkably, so it is essential for the administrative sector to regulate the supply chain of healthcare service goods. There are notable examples of administrative sector’s intervention, such as the intervention of governments and private firms in health aspects to improve nutrition behavior [4], the intervention of donors in malaria drugs to encourage the channel to improve access to these drugs [5], the intervention of the public sectors in a two-tier health system to maximize the total weighted patient welfare [6], and the intervention of the government in demand and supply sides to investigate the relative effectiveness for the influenza vaccine supply chain [7]. For the above cases, the key role of an administrative sector is to design an intervention mechanism from perspective of social benefits. Then it is crucial and indispensable for us to establish an intervention mechanism based on social welfare maximization.

In this paper, we consider a research problem that regulates the supply chain system for healthcare service goods by an intervention mechanism, and the supply chain system is composed of a product provider and an administrative sector. In recent years, the rapid growth of China's economy has improved people’s living standards dramatically, but conventional hospital resource is not enough to meet the residents’ demand expectation in China. Promoting healthcare service goods is an important task of deepening the reform of the medical and health system, and many local governments are promoting healthcare service goods through subsidy strategy. In China, the administrative sector refers to the health commission, the commission of development and reform, the finance department, the civil administration department, the department of human resource and social security and the government department. For an administrative sector, the objective of the intervention mechanism is to maximize social welfare utility instead of maximizing expected profit. Then we should incorporate the objective into the supply chain system when designing the intervention mechanism based on social welfare maximization. Two intervention strategies can be applied to the intervention mechanism for an administrative sector, one strategy is to invest its capital in the demand-growth strategy, such as media publicity, physician training, demand investigation and direct development. Another strategy is to provide rebates or subsidies for the customers. In practice we have to consider the customers’ ability of their willingness to pay, so rebates or subsidies play a key role in making the goods more extensive and attractive [8]. We can easily find that intervention mechanism research of healthcare service goods in the literature, for example, Juergen et al. design an intervention strategy to achieve a higher efficiency of the payoff and production of the public goods including healthcare goods [9], Hamed Mamani et al. consider a subsidy that leads to a socially efficient level of coverage, and derive a tax-subsidy combination that is revenue neutral, but achieves the same effect [10]. Yoko Ibuka et al. find that an increase in the subsidy amount by 1,000 yen (10 USD) leads to a one percentage point increase in the vaccination rate among the elderly in Japan [11]. In our paper, a joint intervention mechanism using both demand-growth strategy and subsidy strategy for healthcare service goods is proposed, so to some extent, the key problem is to optimalize the allocation of the administrative sector’s budget between demand-growth strategy and subsidy strategy.

Bilevel programming is used to obtain the optimal allocation of the administrative sector’s budget between the two intervention strategies. In fact, bilevel programming can be regarded as a particular class of hierarchical mathematical program [12, 13]. In a bilevel programming problem, the objective function of the upper-level problem is identified as the upper-level function, similarly, the objective function of the lower-level problem is identified as the lower-level function. The lower-level variables are constrained to be the solution of the lower-level problem, whereas the remaining variables are regarded as the upper-level variables and parametrize the lower-level problem [14]. In general, a bilevel programming problem contains two levels of optimization tasks [15], the upper-level makes optimal decision based on his objective first, then the lower-level chooses his optimal decision given the upper-level’s action [16,17]. In our paper, the administrative sector is the upper-level decision maker whose objective is to attain social utility maximization, whereas the product provider is the lower-level decision maker whose objective is to attain expected profit maximization, so it is scientific and reasonable to use bilevel programming model. More specifically, the administrative sector determines the optimal budget allocation between demand-growth strategy and subsidy strategy, then the product provider determines the order quantity. The response functions of investment in demand-growth strategy are usually supposed to be concave [18–20]. In other words, expected demand will increase with the money investment in demand-growth strategies. Finally, we explore a specific case study to demonstrate the effectiveness of our model.

The rest of the paper is organized as follows. In Section 2, we provide a literature review related to our research problem. In Section 3, we establish its mathematical formulation according to the research problem. In Section 4, we explore two benchmark approaches. In Section 5, we present the special case. In Section 6, we conduct numerical analysis to obtain the key results. In Section 7, we summarize conclusion remarks and highlight future directions of this research.

## Literature review

In this paper, we consider a research problem that regulates the supply chain system for healthcare service goods by an intervention mechanism, and the supply chain system is composed of a product provider and an administrative sector, the objective of this paper is to facilitate the healthcare service goods to be adopted widely by establishing an intervention mechanism based on social welfare maximization. Therefore in this section, we outline four streams of literature according to the research problem.

First, the paper is related to the economics literature studying contract design for healthcare service goods and other public-interest goods. Regarding the contract design literature of healthcare service goods, Hamed Mamani et al. [21] propose a contract mechanism to reduce the inefficiency in the allocation of influenza vaccines. The proposed contract based on epidemic model reduces the overall financial burden of infection globally and improves the total number infected by seasonal influenza outbreaks. N. Shamsi G. et al. [22] develop a specific option contract for proactively provisioning required vaccine doses from two suppliers (a main and a backup). For the model in this paper, its aim is to minimize the procurement and social costs using the SIR epidemic model. In addition, there are some literature studying contract design for other public-interest goods. Wenhui Zhou et al. [23] discuss two types of contracts that specify the subsidy for energy-saving products with the government’s budget constraint, and the optimal design of the contracts is given under two government objectives: minimizing the total energy consumption and minimizing the average energy consumption. Fei Ye et al. [24] design a coordination contract in a random yield environment to ensure the sustainability of biofuel production and improve the performance of the biofuel supply chain, then over-production risk-sharing contract, under-production risk-sharing contract and mixed contract are examined. Zhaofu Hong et al. [25] study several cooperation contracts for a green product supply chain, the environmental responsibilities of a manufacturer and a retailer are considered in a two-echelon supply chain, the result shows that the cooperation between the manufacturer and the retailer may not always profitably benefit all partners. Given the characteristic of healthcare service goods in China, it is more appropriate to use intervention mechanism for healthcare service goods.

Second, the paper is related to the stream of operations research dealing with intervention mechanism of healthcare service goods and other public-interest goods. Regarding health-related products, Dan Yamin et al. [26] establish an epidemiological game model to find the optimal incentive for vaccination and the expected vaccination coverage. Elodie Adida et al. [27] consider how a socially optimal vaccine coverage can be induced through the central policy-maker’s subsidy to both consumers and the vaccine manufacturer. In fact, subsidy is a frequently-used intervention method. Some papers find a positive correlation between the subsidy and the production. For example, Huaying Gu et al. [28] investigate a electric vehicle manufacturer's optimal production strategy under subsidy and battery recycling when the market demand is uncertain. The results indicate that increased subsidy promotes the electric vehicle manufacturer's optimal production quantity and expected utility. Chunlin Luo et al. [29] consider two manufacturers in a symmetric duopoly setting how to produce the traditional and public interest products under a government’s subsidy scheme, and the result shows that a higher subsidy can increase the sale of the public-interest product. Maxime C. Cohen et al. [30] indicate that government subsidies offered directly to consumers impact the manufacturer’s production, but the policy makers should attach importance to demand uncertainty when designing consumer subsidies, otherwise they will not attain the expected adoption target level. Furthermore, Chaogai Xue et al. [31] study the decision-making of government subsidy on supply chain for straw power generation, and discuss the changes of members’ profits and supply chain’s profits under different subsidy circumstances. Bo Li et al. [32] consider which subsidy strategy is more efficient for environmental-friendly products in a dual-channel supply chain. Different from this stream of literature, we consider demand-growth and subsidy simultaneously.

Third, the paper is broadly related to ever-increasing literature that studies multi-echelon supply chain problem. As a matter of fact, two-echelon and three-echelon are common multi-echelon types of supply chain. For two-echelon supply chain problem, Yi Yuyin et al. [33], Dua Weraikat et al. [34], R.B.O. Kerkkamp et al. [35] and T. Maiti et al. [36] are some good examples. All these above studies consider a two-echelon supply chain model that is comprised of one manufacturer/supplier and one retailer. These studies indicate that both tax and subsidy policies can facilitate the sustainability of the supply chain, furthermore, designing a cooperation mechanism between the manufacturer and the retailer has an important effect on the supply chain. For three-echelon supply chain problems, B.C. Giri et al. [37] and Jian Li et al. [38] are the two examples that studies three-echelon supply chain that is comprised of a supplier, a manufacturer and a retailer. Different coordination strategies and detailed analyses are discussed. The paper of Songsong Liu et al. [39] study the optimal profit distribution in the supply chain consisting of active ingredient plants, formulation plants and markets. Y.N. Wu et al. [40] consider three levels of information sharing in a three-echelon supply chain consisting of a manufacturer, a distributor, and a retailer, and then derive the optimal inventory policy under each level of information sharing. Compared to this stream of literature, our paper considers a common two-echelon healthcare service goods supply chain setting composed of a product provider and an administrative sector.

Forth, there is a stream of literature of bilevel programming research. Walter J. Gutjahr et al. [41], Saemeh Aghajani et al. [42], Yue Zheng et al. [43] and S.M. Alizadeh et al. [44] introduce a bilevel programming model to solve the problem. In general, bilevel programming model is identified as an efficient mathematic method to solve the hierarchical decision-making problem with two different decision objectives. In the bilevel programming model, the decision-maker at the upper level optimizes his/her objective function under a set of constraints first, and then the decision-maker at the lower level optimizes his/her objective function taking into consideration of the upper decision-maker’s action [45]. In our paper, the administrative sector is the upper-level decision maker whose objective is to attain social utility maximization, whereas the product provider is the lower-level decision maker whose objective is to attain expected profit maximization.

In summary, although the above literature have enriched our understanding of the impact of intervention mechanism on supply chain or procurement, the existing literature has not studied intervention mechanism that maximizes social welfare for healthcare service goods, so solving this problem is important and crucial to facilitate the healthcare service goods to be adopted widely. To the best of our knowledge, Ece Zeliha Demirci, Lulu Shao and Huiping Ding [46–48] are closet to our research in supply chain and mechanism design. Demirci and Erkip [46] study the intervention problem for public-interest goods by using bilevel programming model, but they do not consider consumer’s willingness behavior. Shao, et al. [47] formulate a utility model composed of a population of consumers who make utility maximizing choices and manufacturers who set an optimal pricing, then optimal subsidies or optimal price discount rates can be found for policy makers. Ding, et al. [48] explores the collaborative mechanism that motivates supply chain firms to collectively invest in environmental technology and produce environmental friendly products. Due to public attribute of healthcare service goods, intervention mechanism should take into account the maximization of social welfare. With the growth of various health demand in China, Chinese government is promoting healthcare service goods by using subsidy strategy, so it is more scientific and reasonable to consider demand-growth strategy and subsidy strategy jointly. Our research work differs from the above three papers in three dimensions: (1) a method to determine the willingness price of healthcare service goods in China; (2) for healthcare service goods, the administrative sector’s budget allocation between demand-growth strategy and subsidy strategy is explored; (3) due to the characteristic of the healthcare service goods in China, an intervention mechanism considering demand distributions based on social welfare maximization is developed.

## Model

In this section, we establish a mathematical model to design a maximizing social welfare intervention mechanism for healthcare service goods in China. The aim of the intervention mechanism is to expand the adoption of the healthcare service goods. The problem for a common setting composed of a product provider and an administrative sector that regulates the supply chain system for healthcare service goods by an intervention mechanism is considered. The main goal of the product provider is to maximize its expected profit, whereas the main goal of the administrative sector is to improve the healthcare service goods’ availability and adoption, hence promoting social welfare.

First, given the fuzziness and uncertainty of evaluation indicators in Table 1, we establish a variable fuzzy set model to get the willingness price, which can help customers buy a healthcare service goods at a lower price that they are willing to pay. To the best of our knowledge, we are the first to study the willingness price for healthcare service goods in China. Then, we formulate a bilevel programming model to study the intervention problem, to some extent, the key problem is to optimalize the allocation of the administrative sector’s budget between demand-growth strategy and subsidy strategy.

**Table 1:** Healthcare service goods perceptive satisfaction evaluation indicator system.

healthcare service goods perceptive satisfaction evaluation indicator system	basic medical care	general clinic therapy
		Chinese medicine clinic therapy
		simple clinic (used to make up a prescription only)
		conventional family diagnosis
		health record maintenance management
	basic public health	hypertension patient management
		hyperglycemia patient management
		hyperlipidemia patient management
		diabetes patient management
		coronary disease patient management
	perceptive value	disease prevention
		health state improvement
		doctor-patient trust

### 3.1 Willingness price

In general, the public-interest goods price that the customers are willing to pay is lower than the cost price of the goods [49]. Let p_w_ and p_c_ denote the willingness price and the cost price respectively, and the willingness price will be divided into five levels, they are 0.85 p_c_, 0.75 p_c_, 0.65 p_c_, 0.55 p_c_, 0.45 p_c_ respectively. The customers always determine the willingness price on the basis of their perceptive satisfaction evaluation of the healthcare service goods. In other words, the level of willingness price depends on the product perceptive satisfaction level, and there is a positive correlation between the level of willingness price and the perceptive satisfaction evaluation. For example, if the product perceptive satisfaction is regarded as the highest level, then the willingness price will be 0.85 p_c_ accordingly.

#### 3.1.1 Perceptive satisfaction evaluation indicator system

The perceptive satisfaction evaluation indicator system of healthcare service goods is established by following the principle of scientificity, systematicness and operability. According to the indicator system connotation and current situation in China, the evaluation indicator system of perceptive satisfaction should include basic medical care, basic public care and perceptive value. The detailed indicator system is formulated in Table 1.

#### 3.1.2 Variable fuzzy set method

The variable fuzzy set method is based on relative difference function, then the subordination relationship between evaluation objects and standard levels can be acquired by subordination information [50]. In the variable fuzzy set model, we suppose that *u* is the arbitrary element of fuzzy set *U*, the arbitrary element *u* has a relative membership degree *μ*_*A*_(*u*) with the attractive interval *A*, *μ*_*A*_(*u*) ∈ [0, 1]. The arbitrary element *u* has a relative membership degree *μ*_*Ac*_(*u*) with the exclusive interval *A*, *μ*_*Ac*_(*u*) ∈ [0, 1]. The arbitrary element’s relative difference coefficient *D*_*A*_(*u*) for the attract interval *A* is as follows:

$$D_{A}(u)=\mu_{A}(u)-\mu_{Ac}(u)$$ (1)

$$\mu_{A}(u)-\mu_{Ac}(u)=1$$ (2)

We can obtain the relative difference coefficient *D*_*A*_(*u*) according to Eqs 8 and 9, relative difference coefficient *μ*_*A*_(*u*) is as follows:

$$\mu_{A}(u)=[1+D_{A}(u)]/2$$ (3)

**Fig 1:**
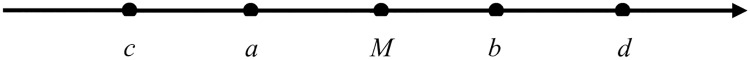
The position relation between zone [*a*, *b*], [*c*, *d*] and point *x*, *M*.

On the continuous membership number axis (Fig 1), we suppose that *X*_0_ = [*a*, *b*] refers to the arbitrary element’s attractive interval, *X* = [*c*, *d*] refers to a range interval including *X*_0_.

In Fig 1, [*c*, *a*] and [*b*, *d*] refer to the arbitrary element’s exclusive interval, *M* refers to the point of relative membership *μ*_*A*_(*u*) = 1 located on attractive interval [*a*, *b*]. We suppose that *x* is the arbitrary point in the interval, if *x* is located on the left of point *M*, its relative difference function is Eq 11; however, if *x* is located on the right of point *M*, its relative difference function is Eq (12).

$$\{\begin{array}{ll} D_{A}\left(u\right)={\left(\frac{x-a}{M-a}\right)}^{\beta } & x\in \left[a,\;M\right]\\ D_{A}\left(u\right)=-{\left(\frac{x-a}{c-a}\right)}^{\beta } & x\in \left[c,\;a\right] \end{array}$$ (4)

$$\{\begin{array}{ll} D_{A}\left(u\right)={\left(\frac{x-b}{M-b}\right)}^{\beta } & x\in \left[M,\;b\right]\\ D_{A}\left(u\right)=-{\left(\frac{x-b}{d-b}\right)}^{\beta } & x\in \left[b,\;d\right] \end{array}$$ (5)

In general, *β* = 1, it means that relative difference function is linear model. Then we can get the relative difference coefficient *μ*_*A*_(*u*) by putting Eqs 4 and 5 into the Eq 3, so single-factor fuzzy matrix *R* is obtained. We suppose that *n* refers to evaluation indicators, *m* refers to evaluation levels, so the variable fuzzy set evaluation model is as follows:

{uh′=[1+(dghdb)α]-1dgh={∑i=1n[ωi(1-μA(u)ih)]p}1pdb={∑i=1n[ωiμA(u)ih)]p}1p (6)

Where, $u_{h}^{\prime }$ is the relative membership degree that is not normalized for level *h*, *h* refers to evaluation degree, *h* = 1, 2,⋯, *m*; *d*_*gh*_ is the generalized weighted distance between the relative membership degree and the left limit point; *d*_*b*_ is the generalized weighted distance between the relative membership degree and the right limit point; *μ*_*A*_(*u*)_*ih*_ refers to the indicator’s relative membership degree for the level *h*; α refers to variable optimization criterion parameter, α = 1,2; *ω*_*i*_ refers to the weight of the evaluation indicator *i*. The relative membership degree $u_{h}^{\prime }$ is normalized as follows:

$$u_{h}=\frac{u_{h}^{\prime }}{\sum_{h=1}^{m}u_{h}^{\prime }}$$ (7)

$$H=\sum_{h=1}^{m}u_{h}\cdot h$$ (8)

Where, *u*_*h*_ refers to the normalized relative membership degree for the level *h*, *H* refers to the evaluation object’s level. The type of willingness price depends on the product perceptive satisfaction level *H*. If the product perceptive satisfaction is regarded as the first level, then the willingness price will be 0.90 p_c_ accordingly.

### 3.2 Intervention mechanism based on bilevel programming

In order to model the problem that regulates the supply chain system for healthcare service goods by means of an intervention mechanism, we assume a hierarchical decision process with two levels of decision. In constructing bilevel programming model for intervention mechanism problem, the following notations in Table 2 will be used.

**Table 2:** Notations used in the bilevel programming model.

Parameters	
*D*	the healthcare service goods’s demand
*P*_*w*_	the willingness price
*P*_*w*_+*s*	the product provider’s revenue from per unit sold
*c*	the cost of each healthcare service goods
*Q*_*T*_	the healthcare service goods’ target amount formulated by the administrative sector
*θ*	the monetary value (RMB) per unit sold
*v*	the salvage value for each unsold goods
*pdf* $f_{B_{d}}(∙)$	the probability density function of demand, and the demand distribution depends on *B*_*d*_
*cdf* $F_{B_{d}}(∙)$	the cumulative distribution function of demand
Decision variables	
*upper level (the administrative sector*)	
*B*	the total budget that is used for intervention mechanism
*B*_*d*_	the budget amount that is allocated to investment in demand-growth strategy
*B*_*s*_	the budget amount that is allocated to subsidy strategy
*s*	the subsidy available to provide for each customer
*lower level (the product provider)*	
*Q*	the quantity amount of healthcare service goods
Objective functions	
*upper level (the administrative sector*)	
*u*(*Q*, *B*_*d*_)-*B*	it refers to social welfare function
*lower leve*l *(the product provider)*	
*E*{*P*(*Q*)|*B*_*d*_}	it refers to the product provider’s expected profit

In our model, the product provider’s problem is similar to a newsvendor problem. The demand distribution depends on the budget amount that is allocated to investment in demand-growth strategy. According to the relevant research in recent years, it is assumed that the cumulative distribution function of demand is monotonously increasing. The monotonicity of $F_{B_{d}}(∙)$ implies that *Q* will increase with the increase of fractile. Especially, we assume that the cumulative distribution function of demand at a given value is a decreasing function of *B*_*d*_, so we can confirm that as *B*_*d*_ increase, so does *Q*.

The bilevel programming model of the intervention mechanism problem is as follows:

$$Model\;I:{\text{max}}_{s,B_{d},B_{s},B}u\left(Q,\;B_{d}\right)-B$$ (9)

$${subject\;to\;B}_{d}+B_{s}\leq B$$ (10)

$$sE[\{\text{min}(Q,\;D){|B}_{d}\}\leq B_{s}$$ (11)

$$s\geq c-P_{w}$$ (12)

s≥0 (13)

$$Q\geq Q_{T}$$ (14)

$$B_{d},\;B_{s},\;Q_{T}\geq 0$$ (15)

$${\text{max}}_{Q}E\left\{P\left(Q\right)\text{|}B_{d}\right\}$$ (16)

where $E\{P(Q)|B_{d}\}=\int_{0}^{Q}[(P_{w}+s)x+v(Q-x)-cQ]f_{B_{d}}(x)d_{x}+\int_{Q}^{\infty }(P_{w}+s-c)Qf_{B_{d}}(x)d_{x}$ refers to the expected profit of the product provider.

In *model I*, Eqs 9 to 16 refer to the administrative sector’s problem, and Eq 16 refers to the product provider’s problem. Eq 10 ensures that the sum of *B*_*d*_ and *B*_*S*_ can’t be higher than the total budget *B*. Eq 11 illustrates that the total subsidy amount is lower than the budget amount allocated to subsidy strategy. Eq 12 indicates that the subsidy per unit product is higher than c − *P*_*w*_, so the profit per unit product is greater than 0. Eq 14 highlights that the product quantity *Q* should be higher than the target amount *Q*_*T*_. In recent years, the Chinese government have attached great importance to the popularization of healthcare service goods, and the government at all levels has formulated clear targets. Eqs 13 and 15 guarantees that *r*, *B*_*d*_, *B*_*r*_, *Q*_*T*_ are non-negative.

In fact, bilevel programming can be regarded as a particular class of hierarchical mathematical program. The upper-level objective function (Eq 9) is identified as the administrative sector’s problem, with the objective of social welfare maximization, while the lower-level objective function (Eq 16) is identified as the product provider’s problem, with the objective of its expected profit maximization. In our paper, we aim to establish an intervention mechanism based on social welfare maximization, and the product provider make an optimal decision under the dominant objective of the administrative sector by establishing a bilevel programming model.

### Remark

We can easily find that Eq 11 can obtain optimal solution only when *sE*[{min(*Q*, *D*)|*B*_*d*_} = *B*_*s*_. In addition to this, it is optimal only when the total budget *B* is equal to the summation of *B*_*d*_ (the budget amount that is allocated to investment in demand-growth strategy) and *B*_*r*_ (the budget amount that is allocated to subsidy strategy).

The expected profit of the product provider *E*[*P*(*Q*)] is concave in *Q* for a given *B*_*d*_, *B*_*s*_ and *s*, which indicates that the product provider has a unique solution. Then we can determine that the administrative sector can achieve his objective by maximizing social welfare due to the uniqueness of *R*[*B*_*d*_, *B*_*s*_, *s*]. *E*[*P*(*Q*)] is concave in *Q* for a given *B*_*d*_, *B*_*s*_ and *s*, which implies that the product provider problem’s solution can be replaced by its first-order condition.

Note that the product provider’s problem is a newsvendor problem essentially, *Model I* can be written as the following single-level mathematical formation:

$$Model\;II:{\text{max}}_{s,B_{d},B_{s},Q}u\left(Q,\;B_{d}\right)-B_{d}-B_{s}$$ (17)

$$subject\;to\;sE[\{\text{min}\left(Q,\;D\right)|B_{d}\}=B_{s}$$ (18)

$$s\geq c-P_{w}$$ (19)

s≥0 (20)

$$Q\geq Q_{T}$$ (21)

$$B_{d},\;B_{s},\;Q_{T}\geq 0$$ (22)

$$F_{B_{d}}(Q)=\frac{P_{w}+s-c}{P_{w}+s-v}$$ (23)

In general, a bilevel programming model is a challenging problem because it is difficult to calculate and obtain optimal solution, however, we obtain an easier solution method by translating a two-level model (*Model I*) into a single-level model (*Model II*). Now *Model II* is a nonlinear program, in which the objective function is not linear.

In the following section, we consider a specific form of the administrative sector’s social welfare function and the mean demand function. In practice, the administrative sector attaches great importance to the number of adopters for healthcare service goods in China, so it is scientific and reasonable to use *θ* times expected sales volume to quantify social welfare, then the social welfare objective function of the administrative sector can be regarded as a linear problem, which is convenient for us to do the following analyses. According to the related literature that studies the relationship between demand and the budget investment in demand-growth strategy *B*_*d*_, we assume that the response function of *B*_*d*_ is increasing and concave, then the mathematic form is as follows:

$$\mu (B_{d})=\mu_{\infty }-\frac{d}{{\left(1+mB_{d}\right)}^{c}}$$ (24)

where, *m*, *d*, *c* > 0.

In Eq 24, we can determine that mean demand has a positive correlation with *B*_*d*_, in other words, if *B*_*d*_ increases, then the mean demand will increase, but with a diminishing rate monotonically. The specific social welfare function is linear form, so we use *θ* times expected sales volume to quantify social welfare, the mathematical program can be written as follows:

$$Model\;III:\;{\text{max}}_{s,B_{d},Q}(\theta -s)Emin\left\{\left(Q,\;D\right)|B_{d}\right\}-B_{d}$$ (25)

$$subject\;to\;F_{B_{d}}(Q)=\frac{P_{w}+s-c}{P_{w}+s-v}$$ (26)

$$s\geq c-P_{w}$$ (27)

$$sE[\{\text{min}\left(Q,\;D\right)|B_{d}\}=B_{s}$$ (28)

$$Q\geq Q_{T}$$ (29)

$$B_{d},\;Q_{T}\geq 0$$ (30)

Next, we analyze specific demand distributions in *model III*. Based on related literature and the actual situation of demand distribution, it is reasonable to consider exponential and lognormal distributions to represent healthcare service goods’ demand distributions induced by *B*_*d*_. Then we consider *Model III*, in which demand distributions depends on *B*_*d*_, and the mean distribution follows Eq 24. We analyze two situations: for the first situation, the variation coefficient is constant; for the second situation, the variation coefficient is related to *B*_*d*_.

### The first situation: The variation coefficient is constant

First, we discuss that the demand distribution is exponential or lognormal when the variation coefficient is constant, and it can be divided into two situations: (*i*) *μ*(*B*_*d*_) for exponential distribution; (*ii*) *μ*(*B*_*d*_) for lognormal distribution, the analysis procedure is as follows:

According to Eq 26, we can obtain the following Eqs 31 and 32,

$$1-F_{B_{d}}(Q)=\frac{c-v}{P_{w}+s-v}$$ (31)

$$Q=F_{B_{d}}^{-1}(\frac{P_{w}+s-c}{P_{w}+s-v})$$ (32)

Then, the expected sales amount *E*[{min(*Q*, *D*)|*B*_*d*_} can be expressed as follows:

$$E[\{\text{min}\left(Q,\;D\right)|B_{d}\}=\int_{0}^{F_{B_{d}}^{-1}(\frac{P_{w}+s-c}{P_{w}+s-v})}xf_{B_{d}}(x)d_{x}+\frac{c-v}{P_{w}+s-v}F_{B_{d}}^{-1}(\frac{P_{w}+s-c}{P_{w}+s-v})$$ (33)

According to Eqs 40 and 32, the administrative sector’s social welfare function can be expressed as follows:

$$u(B_{d},\;s)=(\theta -s)\{\int_{0}^{F_{B_{d}}^{-1}(\frac{P_{w}+s-c}{P_{w}+s-v})}xf_{B_{d}}(x)d_{x}+\frac{c-v}{P_{w}+s-v}F_{B_{d}}^{-1}(\frac{P_{w}+s-c}{P_{w}+s-v})\}-B_{d}$$ (34)

Next, we analyze the above equation’s first order condition in regard to *s*, the mathematical formulation can be written as follows:

$$\begin{array}{r} \frac{\partial u\left(B_{d},s\right)}{\partial s}=\\ -\left\{\int_{0}^{F_{B_{d}}^{-1}\left(\frac{P_{w}+s-c}{P_{w}+s-v}\right)}xf_{B_{d}}\left(x\right)d_{x}+\frac{c-v}{P_{w}+s-v}F_{B_{d}}^{-1}\left(\frac{P_{w}+s-c}{P_{w}+s-v}\right)\right\}+\\ \left(\theta -s\right)\{F_{B_{d}}^{-1}\left(\frac{P_{w}+s-c}{P_{w}+s-v}\right)f_{B_{d}}\left[F_{B_{d}}^{-1}\left(\frac{P_{w}+s-c}{P_{w}+s-v}\right)\right]\frac{1}{f_{B_{d}}\left[F_{B_{d}}^{-1}\left(\frac{P_{w}+s-c}{P_{w}+s-v}\right)\right]}\frac{c-s}{{\left(P_{w}+s-v\right)}^{2}}+\frac{-\left(c-v\right)}{{\left(P_{w}+s-v\right)}^{2}}F_{B_{d}}^{-1}\left(\frac{P_{w}+s-c}{P_{w}+s-v}\right)+\\ \;\frac{c-v}{P_{w}+s-v}\frac{1}{f_{B_{d}}\left[F_{B_{d}}^{-1}\left(\frac{P_{w}+s-c}{P_{w}+s-v}\right)\right]}\frac{{\left(c-v\right)}^{2}}{{\left(P_{w}+s-v\right)}^{2}}\}=0 \end{array}$$ (35)

We can obtain the following mathematical formulation by simplifying Eq 35, the mathematical formulation can be written as follows:

$$\int_{0}^{F_{B_{d}}^{-1}(\frac{P_{w}+s-c}{P_{w}+s-v})}xf_{B_{d}}(x)d_{x}+\frac{c-v}{P_{w}+s-v}F_{B_{d}}^{-1}(\frac{P_{w}+s-c}{P_{w}+s-v})=\frac{\theta -s}{f_{B_{d}}[F_{B_{d}}^{-1}(\frac{P_{w}+s-c}{P_{w}+s-v})]}\frac{{\left(c-v\right)}^{2}}{{\left(P_{w}+s-v\right)}^{3}}$$ (36)

1. We assume that *μ*(*B*_*d*_) follows exponential distribution, the theorem of exponential distribution in mathematical formulation form can be written as follows:
  $$F\left(x\right)=\{\begin{array}{ll} 1-e^{-\gamma x} & x\geq 0\\ 0 & x<0 \end{array}$$ (37)
  Hence,
  $$e^{-\gamma x}=1-F(x)\overset{}{\Rightarrow }\text{ln}\left(e^{-\gamma x}\right)=\text{ln}\left(1-F(x)\right)$$ (38)
  So,
  $$x=-\frac{1}{\gamma }\text{ln}\left(1-F(x)\right)$$ (39)
  Note that
  $$\frac{1}{\gamma }=\mu (B_{d}),1-F(x)=\frac{c-v}{P_{w}+s-v}$$ (40)
  We can obtain the following mathematical formulation by substituting Eq 40 into Eq 36, it can be written as follows:
  $$\mu (B_{d})\frac{c-v}{P_{w}+s-v}\text{ln}(\frac{c-v}{P_{w}+s-v})+\mu (B_{d})\frac{P_{w}+s-c}{P_{w}+s-v}-\mu (B_{d})\frac{c-v}{P_{w}+s-v}\text{ln}(\frac{c-v}{P_{w}+s-v})=(\theta -s)\mu (B_{d})\frac{c-v}{{\left(P_{w}+s-v\right)}^{2}}$$ (41)
  Then, Eq 49 is obtained by simplifying Eq 41,
  $$s^{2}+2(P_{w}-v)s+(\theta v+cv-P_{w}c-P_{w}v-\theta c)=0$$ (42)
  The solution of the above quadratic equation with one unknown is as follows:
  $$s=-P_{w}+\sqrt{P_{w}+\theta (c-v)+P_{w}(v-P_{w})+c(P_{w}-v)}$$ (43)
  For Eq 43, it is obvious to find that the optimal subsidy for each customer is independent of *B*_*d*_ for exponential distribution.
2. We assume that *μ*(*B*_*d*_) follows lognormal distribution, the analysis procedure is similar to Eqs 31 to 43. It can be expressed that the optimal subsidy for each customer is independent of *B*_*d*_ for lognormal distribution when the variation coefficient is constan**t**.

For the first situation, we can find that the optimal subsidy for each customer is independent of the budget amount that is allocated to investment in demand-growth strategy and mean demand when the demand distribution is exponential or lognormal. It is interesting that the fact under the first situation is different from the traditional idea, maybe a large number of people would hold the idea that the optimal subsidy for each customer is related to the planning stage. In other words, the optimal fractile value is constant for the product provider, which means that there is no relation between the optimal fractile and the demand parameter *μ*(*B*_*d*_).

### The second situation: The variation coefficient is related to *B*_*d*_ for lognormal distribution

For the second situation, we assume that the variation coefficient is related to *B*_*d*_ for lognormal distribution. To be more specific, if *B*_*d*_ increases, then the variation coefficient will decrease. This assumption is in accordance with relevant literature and practical situations. Similar to Eq 24, the mathematical formulation for the variation coefficient of the lognormal distribution can be written as follows:

$$cv(B_{d})={cv}_{min}+\frac{{cv}_{eli}}{{\left(1+mB_{d}\right)}^{c}}$$ (44)

Where, *m*, *c* > 0, we assume that if *B*_*d*_ (the demand-growth strategy) increases, then the variation coefficient will decrease, but with a monotonically diminishing rate. *cv*_*eli*_ implies that a portion of variation coefficient can be eliminated. Eq 44 indicates that the lognormal distribution for healthcare service goods approaches a limited distribution with a mean of *μ*_∞_ and a variation coefficient of *cv*_*min*_ as *B*_*d*_ (the demand-growth strategy) approaching infinity.

Next, we need to analyze the dependence relationship between the optimal subsidy for each customer and *B*_*d*_ (the demand-growth strategy) considering the *model III*, the mathematic formulation of mean demand (Eq 24) and the mathematic function of variation coefficient (Eq 37). The specific analyses procedure is similar to that of Eqs 31 to 43. Specifically, it manifests that there is dependence relationship between the optimal subsidy and *B*_*d*_ (the demand-growth strategy). In other words, variability coefficient will decrease as dependence relationship between the optimal subsidy for each customer and *B*_*d*_ (the demand-growth strategy) increases, which means that the optimal subsidy for each customer turns into a mathematic function of *B*_*d*_, that is to say, the optimal subsidy is relevant to the planning stage that can’t be ignored.

## Benchmark approaches

In this section, we introduce two benchmark approaches of intervention mechanism that are general in practice. These two benchmark approaches are used to assess the performance of our proposed model. The first benchmark approach only consider the subsidy that is only determined by the administrative sector, and for the second benchmark approach, the optimal subsidy for each customer is independent of customer demand. Different from these two benchmark approaches, the optimal subsidy of the intervention mechanism is determined by the customer and the product provider jointly in this paper.

### 4.1 Benchmark approach 1

For benchmark approach 1, the subsidy is only determined by the administrative sector. In other words, decisions about intervention tools are not made by the administrative sector and the product provider jointly. In addition, *B*_*d*_ (the demand-growth strategy) is not considered in benchmark approach 1. The aim of benchmark approach 1 is to analyze how the healthcare service goods supply chain system operates with a preset subsidy amount only, in other words, benchmark approach 1 does not consider the effect of demand-growth strategy on supply chain system for healthcare service goods. The mathematic formulation of benchmark approach 1 is written as follows:

$$Benchmark\;approach\;1:\;(\theta -s)min(Q,\;D)-B_{s}$$ (45)

$$subject\;to\;F(Q)=\frac{P_{w}+s-c}{P_{w}+s-v}$$ (46)

$$sEmin(Q,D)=B_{s}$$ (47)

### 4.2 Benchmark approach 2

For the first situation, we can find that the optimal subsidy for each customer is independent of the budget amount that is allocated to investment in demand-growth strategy; whereas for the second situation, there is dependence relationship between the optimal subsidy for each customer and *B*_*d*_, which means that the optimal subsidy for each customer turns into a mathematic function of *B*_*d*_, that is to say, the optimal subsidy is relevant to the planning stage that can’t be ignored. For benchmark approach 1, we consider the subsidy that is preset only by the administrative sector and *B*_*d*_ (the demand-growth strategy), but the decisions are not made jointly. The subsidy may be optimal in the first situation, but it is not optimal in the second situation. The aim of benchmark approach 2 is to analyze how the healthcare service goods supply chain system operates with *B*_*d*_ (the demand-growth strategy) and a preset subsidy amount that is determined by the administrative sector only, not determined by the administrative sector and the product provider jointly. The mathematic formulation of benchmark approach 2 is written as follows:

$$Benchmark\;approach\;2:{\text{max}}_{B_{d},B_{s},Q}\left(\theta -s\right)Emin\left(Q,D\right)-\left(B_{d}+B_{s}\right)$$ (48)

$$subject\;to\;sE\{\text{min}(Q,\;D)|B_{d}\}\leq B_{s}$$ (49)

$$F_{B_{d}}(Q)=\frac{P_{w}+s-c}{P_{w}+s-v}$$ (50)

## Case study

In this section, we introduce the Wudang personalized health package to be served as our case study. Apparently, personalized health package belongs to healthcare service goods.

Wudang is located in the south of China. The case and related data derive from the information center of this district. In 2014, Nation Health Commission of China announced that it would regard Wudang District as pilot zone of basic health reform, so Wudang personalized health package can be a representation example. As is known, conventional hospital resource is not enough to meet the residents’ expectation, so personalized health package provided by related health corporations, community health center and other entities is important to meet the residents’ increasing health demand. In the light of personalized health package’s important implications on the residents’ health and happiness, it has received great attention from government departments, corporation entitities and research institutes. Especially, all levels of governments pay high attention to the promotion of personalized health package, and plenty of government departments have expressed explicit target of personalized health package. In fact, Wudang District faces a dilemma that limit the promotion of personalized health package, furthermore, it does not achieve the target.

In this paper, we aim to establish an intervention mechanism to promote the social welfare. Wudang personalized health package include wearable device, tradition Chinese medicine service package, physician service, tele-medicine and fitness product for each customer. Under the current policy, the government of Wudang District have provided subsidies for personalized health package. A recent personalized health package survey conducted by a local institute implies that 69% of the customers regard subsidy as a key factor when purchasing personalized health package. The budget amount that is allocated to investment in demand-growth strategy including the media publicity, the physician training, the demand investigation and the development, which is expected to attract more customers and improve the efficiency of personalized health package. Unfortunately, the district faces a development dilemma and the performance of current intervention mechanism is not efficient. So we introduce an intervention mechanism, in which the decision is determined by the administrative sector and the product provider jointly.

## Numerical analysis

In Wudang District, the price of personalized health package that the customers are willing to pay is lower than the product’s cost price. Let p_w_ denotes the willingness price, and the willingness price will be divided into five types, they are 0.85 p_c_, 0.75 p_c_, 0.65 p_c_, 0.55 p_c_, 0.45 p_c_ respectively. In general, the customer determines the willingness price on the basis of his/her perceptive satisfaction evaluation for the personalized health package. In other words, the type of willingness price depends on the product perceptive satisfaction level. Based on Eqs 1 to 8, we calculate that *H* = 3, which implies that *P*_*w*_ = 0.65 p_c_.

### 6.1 Basic parameters

We use exponential distribution and lognormal distribution to express personalized health package demand and conduct numerical analysis respectively. Personalized health package demand is expected to follow exponential distribution in the early stage, and personalized health package demand is expected to follow lognormal distribution in the subsequent stage, the demand distribution is in accordance with the practice in China. We consider the mean demand function given in Eq 24, and the administrative sector’s social welfare function given in Eq 25.

We collect relative data from the government information center of Wudang District and actual investigation. For the Wudang personalized health package, the cost of each healthcare service goods is RMB369, then *P*_*w*_ = 0.65, *P*_*c*_ = RMB239.8, the top subsidy for each customer who purchase personalized health package product is RMB145, *θ* = BMB591, *v* = RMB155. We consider the basic period refers to the time range from 2015 to 2017, and the long period refers to the time range from 2015 to 2023. The basic period situation can be seen in Tables 3 and 4, whereas the long period situation can be seen in Tables 5 and 6. For each table, it includes types of intervention mechanism, social welfare, expected profit, subsidy for each customer, expected sales, subsidy amount, and quantity. We use the KNITRO 9.0 Software to calculate the solution of the nonlinear problem.

**Table 3:** The solutions of the basic period given in exponential distribution.

Intervention mechanism	Social welfare (× 10^5^)	*E*[*P*(*Q*)] (× 10^5^)	*s*	Expected sales (× 10^3^)	*B*_*d*_	*B*_*s*_ (× 10^3^)	*μ*(*B*_*d*_) (× 10^3^)	*Q* (× 10^3^)
JM	13.9	10.0	139	2.91	0	39.6	21.3	3.02
Ben. 1	13.6	9.6	139	2.90	0	45.4	21.3	3.11
Ben. 2	13.6	9.6	139	2.90	0	45.4	21.3	3.11

**Table 4:** The solutions of the basic period given in lognormal distribution.

	Intervention mechanism	Social welfare (× 10^5^)	*E*[*P*(*Q*)] (× 10^5^)	*s*	Expected sales (× 10^3^)	*B*_*d*_ (× 10^5^)	*B*_*s*_ (× 10^3^)	*μ*(*B*_*d*_) (× 10^3^)	*Q* (× 10^3^)
*cv* = 0.8	JM	23.26	19.35	133	4.59	2.32	39.6	12.8	4.52
	Ben. 1	17.91	18.56	149	4.32	0	45.4	11.3	4.63
	Ben. 2	19.29	17.26	145	4.13	1.92	45.4	10.1	4.21
*cv* = 1.0	JM	21.13	18.35	133	4.26	2.01	37.1	12.3	4.18
	Ben. 1	15.10	15.12	149	4.11	0	42.5	11.0	4.29
	Ben. 2	17.03	16.98	145	3.60	1.65	42.5	9.81	3.91
*cv* = 1.2	JM	19.89	17.91	133	4.12	1.95	36.9	10.91	4.01
	Ben. 1	13.55	15.39	149	3.99	0	40.11	9.82	4.15
	Ben. 2	16.30	14.99	145	3.45	1.32	40.09	8.62	4.32

**Table 5:** The solutions of the long period given in exponential distribution.

Intervention mechanism	Social welfare (× 10^5^)	*E*[*P*(*Q*)] (× 10^5^)	*s*	Expected sales (× 10^3^)	*B*_*d*_	*B*_*s*_ (× 10^3^)	*μ*(*B*_*d*_) (× 10^3^)	*Q* (× 10^3^)
JM	112	82	136	23	16.1	322.6	161.3	24.1
Ben. 1	110	78	136	25	0	345.4	161.3	25.1
Ben. 2	109	78	136	25.1	11.2	345.4	161.3	25.2

**Table 6:** The solutions of the long period given in lognormal distribution.

	Intervention mechanism	Social welfare (× 10^5^)	*E*[*P*(*Q*)] (× 10^5^)	*s*	Expected sales (× 10^3^)	*B*_*d*_ (× 10^5^)	*B*_*s*_ (× 10^3^)	*μ*(*B*_*d*_) (× 10^3^)	*Q* (× 10^3^)
*cv* = 0.8	JM	238.9	213.35	133	56.9	20.2	320.6	161.8	52.2
	Ben. 1	195.1	188.46	149	45.2	0	325.4	131.3	46.3
	Ben. 2	205.9	201.26	145	47.3	16.3	325.4	160.1	49.1
*cv* = 1.0	JM	185.3	158.95	133	50.3	18.5	317.1	155.3	48.8
	Ben. 1	132.0	138.92	149	41.1	0	292.7	121.0	43.9
	Ben. 2	157.3	136.18	145	43.2	15.5	301.5	153.9	45.1
*cv* = 1.2	JM	149.9	139.61	133	50.2	14.1	310.9	160.7	49.1
	Ben. 1	118.5	99.59	149	37.9	0	270.1	120.2	38.5
	Ben. 2	126.3	114.25	145	40.5	10.9	289.9	149.2	45.2

### 6.2 Result analysis

Tables 3 to 6 imply that demand uncertainty has an important impact on the social welfare value, then we discuss three specific aspects: (1) subsidy for each customer; (2) social welfare improvement compared with the two benchmark approaches; (3) expected profit improvement compared with the two benchmark approaches.

(1) First, we analyze the subsidy variation between joint mechanism and two benchmarks, the result can be seen in Table 7.

**Table 7:** Subsidy variation between three mechanisms.

*cv*	Basic period		Long period	
	Exponential distribution	Lognormal distribution	Exponential distribution	Lognormal distribution
0.8	139	133	136	133
1.0	139	149	136	144
1.2	139	145	136	145
  In Table 7, we can find that the subsidy is optimal under the exponential situation, but it is not optimal under the lognormal situation, because the optimal subsidy for each customer turns into a mathematic function of *B*_*d*_ when the demand follows lognormal distribution.
(2) Next, we analyze the social welfare improvement compared to the two benchmark approaches, the result can be seen in Table 8.

**Table 8:** Social welfare improvement compared to two benchmark approaches.

*cv*	Basic period		Long period	
	Exponential distribution (Ben.1, Ben.2)	Lognormal distribution (Ben.1, Ben.2)	Exponential distribution (Ben.1, Ben.2)	Lognormal distribution (Ben.1, Ben.2)
0.8	0.02%	29.8%, 20.6%	0.18%	22.4%, 16.0%
1.0		39.0%, 24.1%		40.3%, 17.8%
1.2		46.8%, 22.0%		26.5%, 18.7%
  In Table 8, we can find that when the demand follows exponential distribution, the welfare difference between the joint intervention mechanism and benchmark approaches is tiny. However, when the demand follows lognormal distribution, the social welfare of the joint intervention mechanism established by us is higher than the two benchmark approaches. Especially, benchmark 1 is current policy, and we find that the social welfare for the joint intervention mechanism has a more apparent improvement than current policy, which implies that our intervention mechanism is effective.
(3) Finally, we analyze the expected profit improvement compared to the two benchmark approaches, the result can be seen in Table 9.

**Table 9:** Expected profit improvement compared to two benchmark approaches.

*cv*	Basic period		Long period	
	Exponential distribution (Ben.1, Ben.2)	Lognormal distribution (Ben.1, Ben.2)	Exponential distribution (Ben.1, Ben.2)	Lognormal distribution (Ben.1, Ben.2)
0.8	4%	4.30%, 12.1%	5%	13.2%, 5.80%
1.0		13.8%, 14.1%		14.4%, 16.7%
1.2		16.4%, 19.5%		37.5%, 20.0%
  In Table 9, we can find that when the demand follows exponential distribution, the expected profit difference between the joint intervention mechanism and benchmark approaches is tiny. However, when the demand follows lognormal distribution, the expected profit of the joint intervention mechanism established by us is higher than the two benchmark approaches. Especially, the expected profit improvement will increase with the increase of *cv*.

## Conclusion

In this paper, we consider a research problem that regulates the supply chain system for healthcare service goods by an intervention mechanism, and the supply chain system is composed of a product provider and an administrative sector. Healthcare service goods belongs to public goods, so we should not regard the expected profit as the sole objective. Different from pre-existing research, we establish a supply chain intervention mechanism based on social welfare maximization for healthcare service goods. In specific, we analyze the relationship between the optimal subsidy for each customer and the *B*_*d*_ (the budget amount that is allocated to investment in demand-growth strategy). We attempt to analyze the problem by using variable fuzzy set method and bilevel programming model.

The first contribution of our study is that the intervention mechanism for healthcare service goods can generate more abundant social welfare than the two benchmark approaches that are used generally in practice. The Wudang personalized health package case study implies that our mathematic model that is applied to intervention mechanism for healthcare service goods is scientific and effective. Compared with the two benchmark approaches, our joint intervention mechanism can help the administrative sector to achieve the target and increase social welfare. The second contribution is that the evaluation model that is used to obtain the willingness piece is pivotal for the intervention mechanism. Furthermore, *B*_*d*_ (the demand-growth strategy) plays a key role in case study. Especially, the optimal subsidy for each customer is a mathematic function of *B*_*d*_ under the second situation. We explore a perceptive satisfaction evaluation indicator system for healthcare service goods, and the indicator system include basic medical care, basic public care and perceptive value mainly. Besides, two intervention strategies composed of demand-growth strategy and subsidy strategy are used to the combination of intervention mechanism jointly.

In fact, tax credit also plays an important role in promoting the adoption of healthcare service goods, but we do not consider tax credit in our paper. As is known, the demand of the healthcare service goods may be influenced by regional policy, educational level, consumption structure and medical service convenience, which should be analyzed deeply in the future.

## Supporting information

S1 File Supporting information file (data). (XLSX)
